# Prevalence of and factors associated with receipt of provider recommendation for influenza vaccination and uptake of influenza vaccination during pregnancy: cross-sectional study

**DOI:** 10.1186/s12884-021-04182-w

**Published:** 2021-10-27

**Authors:** Alexandra Brixner, Susanne Brandstetter, Merle M. Böhmer, Birgit Seelbach-Göbel, Michael Melter, Michael Kabesch, Christian Apfelbacher, KUNO-Kids study group

**Affiliations:** 1 University Children’s Hospital Regensburg (KUNO-Clinics), at the Hospital St. Hedwig of the Order of St. John, University of Regensburg, Regensburg, Germany; 2 Member of the Research and Development Campus Regensburg (WECARE) at the Hospital St. Hedwig of the Order of St. John, Regensburg, Germany; 3 Bavarian Health and Food Safety Authority, Oberschleissheim, Germany; 4 Institute of Social Medicine and Health Systems Research, Otto von Guericke University Magdeburg, Magdeburg, Germany; 5 Clinic of Obstetrics and Gynaecology St. Hedwig, University of Regensburg, Regensburg, Germany

**Keywords:** Influenza, Vaccine, Pregnancy, Uptake, Recommendation

## Abstract

**Background:**

Seasonal influenza vaccination has been recommended for pregnant women in Germany since 2010. The aim of this study was to examine prevalence and determinants of receipt of provider recommendation for influenza vaccination as well as influenza vaccination uptake during pregnancy.

**Methods:**

We analysed data from the “KUNO Kids Health Study”, a prospective birth cohort. During the study period (5th July 2015 to 27th June 2018) data were collected from participating mothers by interview and questionnaire. According to Andersen’s behavioural model of health services use potential influencing factors describing the circumstances and characteristics of the mothers and their pregnancies which are potentially affecting whether women receive a recommendation for a vaccination or whether they utilize influenza vaccination were classified into three domains: ‘predisposing characteristics’, ‘enabling resources’ and ‘need’. Using multivariable logistic regression models odds ratios (OR) and corresponding 95% confidence intervals (95% CI) were calculated.

**Results:**

As a combined result across three flu seasons, 368 of 1814 (20.3%) women received an influenza vaccination recommendation during pregnancy. Having had a high-risk pregnancy increased the odds of receiving a vaccination recommendation (OR = 1.3; 95% CI = 1.0–1.6; *p* = 0.045). In contrast, pregnancy onset in summer (OR = 0.7; 95% CI = 0.5–1.0; *p* = 0.027), autumn (OR = 0.4; 95% CI = 0.3–0.5; *p* < =0.001) or winter (OR = 0.5; 95% CI = 0.3–0.6; *p* < =0.001) (compared to spring) as well as mother’s birthplace outside Germany (OR = 0.6; 95% CI = 0.4–0.9; *p* = 0.023) reduced the chance of getting a vaccination recommendation.

Two hundred forty-two of one thousand eight hundred sixty-five (13%) women were vaccinated against influenza during pregnancy. Having received a vaccination recommendation was strongly associated with vaccination uptake (OR = 37.8; 95% CI = 25.5–55.9; *p* < =0.001). Higher health literacy status was also associated with a higher chance of vaccination uptake (OR = 1.7; 95% CI = 1.2–2.6; *p* = 0.008), whereas pregnancy onset in autumn (compared to spring) reduced the chance (OR = 0.5; 95% CI = 0.3–0.8; *p* = 0.008).

**Conclusions:**

At 13% the uptake rate of influenza vaccination is low. Having received a recommendation to vaccinate was strongly associated with uptake but only one fifth of all mothers report such a recommendation. Raising awareness in physicians regarding vaccinating during pregnancy seems to be of essential importance to increase vaccine uptake and to prevent influenza-related complications in pregnant women.

**Supplementary Information:**

The online version contains supplementary material available at 10.1186/s12884-021-04182-w.

## Background

Pregnant women are at increased risk for influenza-associated complications like pneumonia and, less commonly, myositis, rhabdomyolysis, encephalitis or myocarditis. This leads to higher rates of hospitalization and increased morbidity and mortality among pregnant women [1–3]. In addition, the unborn child is in danger of being affected by maternal influenza infection, potentially leading to preterm delivery, spontaneous abortion and congenital anomalies [4–8].

Influenza vaccination is available to and approved for pregnant women. Not only does it protect pregnant women from influenza infection, but the vaccination also benefits the child. Maternal immunization is associated with a reduced rate of preterm birth and a reduced rate of suboptimal birth weight [9–12]. Via placental transfer of maternally-derived antibodies nest protection leads to a reduced risk of influenza illness in infants under 6 months of age, who are too young to be vaccinated themselves [13, 14]. The fact that influenza vaccination is not licensed for children under the age of 6 months stresses the importance of maternal immunization during pregnancy.

Seasonal influenza vaccination has been recommended for pregnant women in Germany since 2010. The recommendations in Germany state that all pregnant women from the 2nd trimester onwards should be vaccinated with the quadrivalent inactivated influenza vaccine, pregnant women with underlying chronic diseases even from the 1st trimester [15, 16].

Although the efficacy [17, 18] and safety [19–22] of influenza vaccination has been shown by many studies, influenza vaccination uptake during pregnancy remained quite low so far, as several studies have shown [23–27]. Possible reasons frequently suggested for low uptake are maternal concerns about the safety of the vaccine to the unborn child, lacking recommendations by health care workers (HCWs) and the perception that the vaccination is not necessary [26–30].

Our study was conducted to examine the current prevalence of receipt of provider recommendation for influenza vaccination as well as influenza vaccination uptake during pregnancy among all participating women in the KUNO Kids study [31]. A further aim of our study was to identify factors affecting receipt of provider recommendation for influenza vaccination and uptake of vaccination. This distinguishes our study from most comparable studies where the focus was mainly on reasons for accepting or rejecting the influenza vaccination named by the pregnant women themselves.

## Materials and methods

### Study design and data collection

Methods and design of KUNO Kids health study have been described in detail before [31]. It is an ongoing prospective birth cohort study conducted at the clinic St. Hedwig in Regensburg, Bavaria, Germany and aims to explore a broad range of factors influencing the development and health of children. Briefly, all mothers giving birth at St. Hedwig hospital in Regensburg were asked to take part in the study within 48 h after birth. After mothers have given informed consent a computer-assisted interview and a self-report questionnaire with the mother were performed. Exclusion criteria were maternal age less than 18 years and insufficient German language skills. Also, women who had already participated in the study with a previous child were not allowed to take part once again [31, 32]. Data from 5th July 2015 to 27th June 2018 were used for the purposes of our study.

### Variables and measurements

#### Outcome variables

To examine the frequency of receipt of provider recommendation for influenza vaccination and uptake rate of influenza vaccination the mothers were asked whether their gynaecologist/obstetrician had advised them to get vaccinated against influenza during pregnancy and whether they received influenza vaccination during pregnancy. The information on vaccination uptake was missing for 21 participants.

#### Predictor variables: predisposing characteristics, enabling resources and need factors

Variables potentially related to receipt of provider recommendation for influenza vaccination or influenza vaccination uptake were chosen according to Andersen’s behavioural model of health services use which distinguished three domains: ‘predisposing characteristics’, ‘enabling resources’ and ‘need’ [33].

##### Predisposing characteristics

- ▪ mother’s age (years), which was collected through interview.
- ▪ country of birth, which was collected through interview.
- ▪ marital status, which was collected through interview.
- ▪ being primipara or multipara, which was collected through interview.
- ▪ highest educational level, which was collected through interview. As a measurement instrument for educational level the “CASMIN” [34] (Comparative Analysis of Social Mobility in Industrial Nations) educational classification was applied in this study. This classification scheme contains nine levels of educations qualification, which can be summarized in three main categories: lowest, medium and highest educational level.
- ▪ occupational status, which was collected through interview.
- ▪ type of employment, which was collected through interview.
- ▪ type of insurance, which was collected through interview.
- ▪ current living space per person, which was collected through interview.
- ▪ healthy lifestyle, which was collected through interview. In this context healthy lifestyle was investigated by the factors body mass index (BMI), sporting activities and healthy eating. According to the WHO criteria [35] women with a BMI < 18,5 kg/m^2^ were thereby classified as underweight, women with a BMI 18,5–24,9 kg/m^2^ as normal weight and women with a BMI > 25,0 kg/m^2^ as overweight. Maintaining a healthy eating was supposed when women stated that they eat fruits and vegetables nearly daily.
- ▪ smokers living in the household, which was collected through interview.
- ▪ strength of social support, which was collected through interview. Information about social support was gained with the help of the questionnaire F-SozU-K14 [36]. Thereby, the mothers were, for instance, asked whether they experience much understanding and security from others or whether they have a person, they can go to when they are feeling depressed.

##### Enabling resources

- ▪ mother’s health literacy, which was collected through interview. Health literacy was assessed with the health care scale (16 items) of the questionnaire HLS-EU-Q47 [37]. The questions refer to an individual’s ability to access, understand, appraise, and apply health information in the field of health care (in contrast to prevention and health promotion*)* and could be answered on a four-point Likert scale from “very easy”, “fairly easy”, “fairly difficult” to “very difficult”.
- ▪ satisfaction with gynaecological/obstetric treatment during pregnancy, which was collected through questionnaire.
- ▪ number of doctor visits during pregnancy, which was collected through questionnaire.
- ▪ claiming of additional prenatal diagnostics, such as amniocentesis, 3D ultrasound or nuchal translucency measurement.
- ▪ For analysing possible influencing factors on vaccination uptake, the receipt of vaccination recommendation by HCW was also considered an enabling resource, which was collected through questionnaire.

##### Need factors

- ▪ having a high-risk pregnancy, which was collected through interview. It describes a situation in which conditions are present which could threaten the life or health of mother or foetus.
- ▪ presence of maternal pre-existing conditions (including allergic rhinitis/conjunctivitis, bronchial asthma, neurodermitis, Crohn’s disease, ulcerative colitis, psoriasis arthritis, rheumatoid arthritis, other autoimmune disease, Diabetes mellitus type 1 or 2, hepatobiliary diseases, kidney disease, thyroid disease, tumour disease, coagulation disorder, heart rhythm disorder, heart attack, high blood pressure, other metabolic disease, multiple sclerosis, epilepsy), which was collected through questionnaire.
- ▪ season of pregnancy onset, which was collected through interview.

### Statistical analysis

Statistical analysis was performed using IBM SPSS Statistics 24. At first, descriptive analyses of the characteristics of the study population were performed. Differences in characteristic features between those who received a recommendation by HCW and those who did not and respectively between those who had received influenza vaccination during pregnancy and those who had not were analysed.

Furthermore, univariable logistic regression analyses were conducted to identify variables related to receipt of provider recommendation for influenza vaccination or uptake of influenza vaccination during pregnancy. Odd Ratios (OR) with 95% confidence intervals (95% CI) and *p*-values were calculated. Variables with *p* < 0.20 in univariable analysis were included in the multivariable logistic regression models.

For analysing trend of receipt of provider recommendation for influenza vaccination and uptake rate of influenza vaccination, percentage rates of each examined season in this study, particularly 2015/2016, 2016/2017 and 2017/2018, were calculated and subsequently compared.

## Results

### Characteristics of the study population

During the study period (5th July 2015 to 27th June 2018) 2620 participants were included in the KUNO Kids study. Of these, 1886 also completed the basic questionnaire and could therefore be considered in further statistical evaluation. This corresponds to more than a 25% loss from the total study enrolment.

Regarding the analytical sample, the median age was 34.33 years (SD = 4.46). 12.2% were born in a country other than Germany, 80.5% were married and living together, 54.8% were primipara. Further characteristics of the study population are shown in Table 1.

**Table 1:** Characteristics of the study population

	Valid values for this variable	
Age (years) (M,SD)	1865	34.33 (4.46)
Country of birth other than Germany (N,%)	1858	226 (12.2)
Marital status (N,%)	1856	
Divorced, widowed, living alone		42 (2.3)
Unmarried and living together		320 (17.2)
Married and living together		1494 (80.5)
Primipara (N,%)	1874	1027 (54.8)
Highest education^a^ (N,%)	1849	
Low		181 (9.8)
Middle		609 (32.9)
High		1059 (57.3)
Occupational status (N,%)	1847	
Not employed		269 (14.6)
Employed less than full-time (part-time, marginally employed, internship etc.)		571 (30.9)
Employed full-time		1007 (54.5)
Insurance (N,%)	1851	
Publicly insured or foreign insurance		1573 (85.0)
Privately insured		278 (15.0)
Living space per person (M,SD)	1817	37.02 (16.6)
Social support (M,SD)	1839	4.43 (0.52)
Healthy lifestyle		
BMI (before pregnancy) (N,%)	1863	
Underweight		45 (2.4)
Normal weight		1132 (60.8)
Overweight		686 (36.8)
Sporting activities (N,%)	1868	
No sporting activities		669 (35.8)
Regularly sporting activities < 1 h/week		204 (10.9)
Regularly sporting activities 1–2 h/week		476 (25.5)
Regularly sporting activities > 2 h/week		519 (27.8)
Healthy eating^b^ (N,%)	1849	860 (46.5)
Smoker in household (N,%)	1852	397 (21.4)
Health literacy (M,SD)	1825	3.14 (0.44)
Receipt of provider recommendation for influenza vaccination (N,%)	1814	368 (20.3)
Satisfaction with gynaecological/obstetric treatment during pregnancy (N,%)	1855	
Very unsatisfied or unsatisfied		66 (3.6)
Satisfied		593 (32.0)
Very satisfied		1196 (64.5)
Number of doctor contacts during pregnancy (M,SD)	1875	4.57 (5.75)
Claiming of additional prenatal diagnostics (N,%)	1818	1453 (79.9)
High-risk pregnancy (N,%)	1846	786 (42.6)
Underlying chronic disease ^c^ (N,%)	1800	967 (53.7)
Season of pregnancy onset (N,%)	1870	
Spring (April, May, June)		437 (23.4)
Summer (July, August, September)		442 (23.6)
Autumn (October, November, December)		539 (28.8)
Winter (January, February, March)		452 (24.2)

^a^According to CASMIN-classification

^b^Nearly daily consumption of fruits and vegetables

^c^Including allergic rhinitis/conjunctivitis, bronchial asthma, neurodermitis, Crohn’s disease, ulcerative colitis, psoriasis arthritis, rheumatoid arthritis, other autoimmune disease, Diabetes mellitus type 1 or 2, hepatobiliary disease, kidney disease, thyroid disease, tumour disease, coagulation disorder, heart rhythm disorder, heart attack, high blood pressure, other metabolic disease, multiple sclerosis, epilepsy

### Receipt of provider recommendation for influenza vaccination

In total, 368 women (20.3%) responded that they had received an influenza vaccination recommendation by HCW during their pregnancy. Results of univariable analyses are presented in Table 2 and revealed country of birth other than Germany and season of pregnancy onset as statistically significant factors associated with vaccination recommendation (*p* < 0.05, compare Table 2).

**Table 2:** Univariable analysis of factors associated with receipt of provider recommendation for influenza vaccination

	Receipt of provider recommendation for influenza vaccination (*N* = 368)	No receipt of provider recommendation for influenza vaccination (*N* = 1446)	OR; 95% CI; p
Age (years) (M,SD)	34.10 (4.79)	34.36 (4.34)	0.987; 0.962–1.013; 0.326
Country of birth other than Germany (N,%)	29 (8.0)	184 (12.9)	0.590; 0.391–0.888; 0.012
Marital status (N, %)			
Divorced, widowed, living alone	6 (1.7)	33 (2.3)	0.696; 0.289–1.677; 0.419
Unmarried and living together	56 (15.6)	251 (17.6)	0.854; 0.622–1.172; 0.329
Married and living together	298 (82.8)	1141 (80.1)	Ref.
Primipara (N,%)	195 (53.6)	795 (55.3)	0.933; 0.741–1.175; 0.557
Highest education ^a^ (N,%)			
Low	30 (8.3)	141 (9.9)	0.840; 0.550–1.281; 0.418
Middle	124 (34.4)	466 (32.8)	1.050; 0.818–1.349; 0.701
High	206 (57.2)	813 (57.3)	Ref.
Occupational status (N,%)			
Not employed	45 (12.6)	216 (15.2)	0.831; 0.581–1.189; 0.311
Employed less than full-time (part-time, marginally employed, internship etc.)	119 (33.2)	429 (30.2)	1.107; 0.856–1.431; 0.439
Employed full-time	194 (54.2)	774 (54.5)	Ref.
Private insurance (N,%)	52 (14.4)	219 (15.4)	0.927; 0.668–1.285; 0.648
Living space per person (M,SD)	36.68 (16.97)	37.30 (16.67)	0.998; 0.991–1.005; 0.534
Social support (M,SD)	4.44 (0.51)	4.43 (0.53)	1.041; 0.834–1.299; 0.720
Healthy lifestyle			
BMI (before pregnancy) (N,%)			
Underweight	10 (2.7)	34 (2.4)	1.179; 0.574–2.424; 0.654
Normal weight	217 (59.6)	870 (61.0)	Ref.
Overweight	137 (37.6)	523 (36.7)	1.050; 0.826–1.335; 0.689
Sporting activities (N,%)			
No sporting activities	129 (35.5)	515 (35.9)	1.002; 0.748–1.342; 0.990
Regularly sporting activities < 1 h/week	40 (11.0)	154 (10.7)	1.039; 0.689–1.567; 0.855
Regularly sporting activities 1–2 h/week	94 (25.9)	364 (25.4)	1.033; 0.754–1.416; 0.840
Regularly sporting activities > 2 h/week	100 (27.5)	400 (27.9)	Ref.
Healthy eating^b^ (N,%)	169 (46.8)	664 (46.9)	0.998; 0.792–1.258; 0.988
Smoker in household (N,%)	79 (21.9)	303 (21.3)	1.038; 0.785–1.374; 0.793
Health literacy (M,SD)	3.15 (0.45)	3.13 (0.44)	1.090; 0.836–1.419; 0.525
Satisfaction with gynaecological/obstetric treatment during pregnancy (N,%)			
Very unsatisfied or unsatisfied	13 (3.6)	53 (3.7)	0.953; 0.511–1.777; 0.880
Satisfied	114 (31.4)	460 (32.2)	0.963; 0.750–1.236; 0.767
Very satisfied	236 (65.0)	917 (64.1)	Ref.
Number of doctor contacts during pregnancy (M,SD)	4.67 (5.68)	4.51 (5.64)	1.005; 0.985–1.025; 0.631
Claiming of additional prenatal diagnostics (N,%)	290 (80.8)	1114 (80.0)	1.049; 0.782–1.407; 0.750
High-risk pregnancy (N,%)	169 (46.9)	586 (41.4)	1.252; 0.992–1.579; 0.058
Underlying chronic disease ^c^ (N,%)	188 (53.7)	740 (53.5)	1.007; 0.796–1.274; 0.955
Season of pregnancy onset (N,%)			
Spring (April, May, June)	122 (33.2)	296 (20.7)	Ref.
Summer (July, August, September)	102 (27.8)	327 (22.9)	0.757; 0.557–1.028; 0.075
Autumn (October, November, December)	67 (18.3)	445 (31.1)	0.365; 0.262–0.509; <=0.001
Winter (January, February, March)	76 (20.7)	363 (25.4)	0.508; 0.367–0.703; <=0.001

^a^According to CASMIN-classification

^b^Nearly daily consumption of fruits and vegetables

^c^Including allergic rhinitis/conjunctivitis, bronchial asthma, neurodermitis, Crohn’s disease, ulcerative colitis, psoriasis arthritis, rheumatoid arthritis, other autoimmune disease, Diabetes mellitus type 1 or 2, hepatobiliary disease, kidney disease, thyroid disease, tumour disease, coagulation disorder, heart rhythm disorder, heart attack, high blood pressure, other metabolic disease, multiple sclerosis, epilepsy

Being born in a country other than Germany, decreased the odds of getting a vaccination recommendation by almost the half (OR = 0.590; 95% CI = 0.391–0.888; *p* = 0.012). Pregnant women who had their pregnancy onset in autumn (OR = 0.365; 95% CI = 0.262–0.509 *p* < =0.001) or winter (OR = 0.508; 95% CI = 0.367–0.703; *p* < =0.001) had significantly lower odds of receiving vaccination recommendation by HCW compared to women whose pregnancy onset was in spring.

Results of multivariable analyses can be seen in Table 4. Birthplace outside Germany reduced the chance of getting a vaccination recommendation (OR = 0.618; 95% CI = 0.408–0.937; *p* = 0.023). Moreover, having a pregnancy onset in summer (OR = 0.704; 95% CI = 0.515–0.962; *p* = 0.027), autumn (OR = 0.350; 95% CI = 0.250–0.490; *p* < =0.001) or winter (OR = 0.460; 95% CI = 0.330–0.642; *p* < =0.001) decreased the chance of getting a vaccination recommendation compared to having pregnancy onset in spring. Additionally, women who had a high-risk pregnancy showed increased odds of getting a vaccination recommendation (OR = 1.275; 95% CI = 1.005–1.618; *p* = 0.045).

### Uptake of influenza vaccination

Out of 1865 women 242 (13.0%) were vaccinated against influenza during pregnancy. Results of univariable analyses are shown in Table 3. In univariable analysis country of birth, health literacy, vaccination recommendation by HCW, high-risk pregnancy and season of pregnancy onset reached statistical significance (*p* < 0.05).

**Table 3:** Univariable analysis of factors associated with uptake of influenza vaccination

	Uptake of influenza vaccination (*N* = 242)	No uptake of influenza vaccination (*N* = 1623)	OR; 95% CI; p
Age (years) (M,SD)	34.67 (4.77)	34.28 (4.39)	1.020; 0.989–1.052; 0.211
Country of birth other than Germany (N,%)	19 (7.9)	203 (12.7)	0.594; 0.363–0.971; 0.038
Marital status (N,%)			
Divorced, widowed, living alone	4 (1.7)	37 (2.3)	0.680; 0.240–1.928; 0.468
Unmarried and living together	32 (13.4)	283 (17.7)	0.711; 0.479–1.055; 0.090
Married and living together	203 (84.9)	1277 (80.0)	Ref.
Primipara (N,%)	132 (55.2)	884 (54.8)	1.019; 0.775–1.339; 0.894
Highest education^a^ (N,%)			
Low	19 (8.0)	160 (10.1)	0.711; 0.429–1.179; 0.186
Middle	69 (29.0)	534 (33.5)	0.774; 0.570–1.049; 0.098
High	150 (63.0)	898 (56.4)	Ref.
Occupational status (N,%)			
Not employed	33 (13.9)	235 (14.8)	0.945; 0.628–1.422; 0.786
Employed less than full-time (part-time, marginally employed, internship etc.)	75 (31.6)	487 (30.6)	1.036; 0.763–1.406; 0.819
Employed full-time	129 (54.4)	868 (54.6)	Ref.
Private insurance (N,%)	41 (17.2)	235 (14.8)	1.203; 0.836–1.730; 0.320
Living space per person (M,SD)	36.08 (15.02)	37.20 (16.87)	0.996; 0.987–1.004; 0.340
Social support (M,SD)	4.46 (0.49)	4.43 (0.53)	1.121; 0.857–1.466; 0.403
Healthy lifestyle			
BMI (before pregnancy) (N,%)			
Underweight	8 (3.3)	37 (2.3)	1.574; 0.718–3.452; 0.257
Normal weight	135 (56.3)	983 (61.4)	Ref.
Overweight	97 (40.4)	582 (36.3)	1.214; 0.917–1.606; 0.176
Sporting activities (N,%)			
No sporting activities	77 (32.1)	585 (36.4)	0.893; 0.629–1.269; 0.529
Regularly sporting activities < 1 h/week	33 (13.8)	168 (10.5)	1.333; 0.847–2.099; 0.214
Regularly sporting activities 1–2 h/week	64 (26.7)	406 (25.3)	1.070; 0.740–1.548; 0.719
Regularly sporting activities > 2 h/week	66 (27.5)	448 (27.9)	Ref.
Healthy eating^b^ (N,%)	109 (45.8)	742 (46.7)	0.966; 0.735–1.269; 0.802
Smoker in household (N,%)	48 (20.2)	348 (21.8)	0.905; 0.645–1.269; 0.561
Health literacy (M,SD)	3.20 (0.45)	3.13 (0.44)	1.483; 1.086–2.026; 0.013
Receipt of provider recommendation for influenza vaccination (N,%)	195 (81.6)	172 (10.9)	36.099; 25.099–51.919; <=0.001
Satisfaction with gynaecological/obstetric treatment during pregnancy (N,%)			
Very unsatisfied or unsatisfied	7 (2.9)	59 (3.7)	0.797; 0.357–1.776; 0.578
Satisfied	78 (32.6)	511 (31.9)	1.025; 0.765–1.373; 0.869
Very satisfied	154 (64.4)	1034 (64.5)	Ref.
Number of doctor contacts during pregnancy (M,SD)	4.93 (6.18)	4.53 (5.70)	1.011; 0.990–1.033; 0.310
Claiming of additional prenatal diagnostics (N,%)	200 (83.7)	1244 (79.7)	1.307; 0.908–1.882; 0.150
High-risk pregnancy (N,%)	121 (50.6)	657 (41.4)	1.452; 1.105–1.906; 0.007
Underlying chronic disease ^c^ (N,%)	130 (55.8)	826 (53.4)	1.102; 0.835–1.454; 0.493
Season of pregnancy onset (N,%)			
Spring (April, May, June)	85 (35.4)	349 (21.7)	Ref.
Summer (July, August, September)	67 (27.9)	371 (23.1)	0.741; 0.521–1.054; 0.096
Autumn (October, November, December)	37 (15.4)	495 (30.8)	0.307; 0.204–0.462; <=0.001
Winter (January, February, March)	51 (21.3)	394 (24.5)	0.531; 0.365–0.774; 0.001

^a^According to CASMIN-classification

^b^Nearly daily consumption of fruits and vegetabels

^c^Including allergic rhinitis/conjunctivitis, bronchial asthma, neurodermitis, Crohn’s disease, ulcerative colitis, psoriasis arthritis, rheumatoid arthritis, other autoimmune disease, Diabetes mellitus type 1 or 2, hepatobiliary disease, kidney disease, thyroid disease, tumour disease, coagulation disorder, heart rhythm disorder, heart attack, high blood pressure, other metabolic disease, multiple sclerosis, epilepsy

Being born in a country other than Germany almost halved the odds of getting influenza vaccination (OR = 0.594; 95% CI = 0.363–0.971; *p* = 0.038). Contrary, increasing health literacy went along with higher odds (OR = 1.483; 95% CI = 1.086–2.026; *p* = 0.013).

Having received a vaccination recommendation by HCW increased the odds around 36-fold (OR = 36.099; 95% CI = 25.099–51.919; *p* < =0.001). 81.6% of all vaccinated women stated that they received a provider recommendation, whereas only 10,9% of unvaccinated women report the receipt of provider recommendation for vaccination. Additionally, mothers diagnosed with high-risk pregnancy showed greater odds of getting influenza vaccination (OR = 1.452; 95% CI = 1.105–1.906; *p* = 0.007). The odds of women with pregnancy onset in winter (OR = 0.531; 95% CI = 0.365–0.774; *p* = 0.001) or autumn (OR = 0.307; 95% CI = 0.204–0.462; *p* < =0.001) was significantly lower compared to women whose pregnancy onset was in spring.

Multivariable analysis showed a significant (*p* < 0.05) association between vaccination status and health literacy, respective season of pregnancy onset and vaccination recommendation by HCW. Results are shown in Table 4. Increasing health literacy went along with a higher chance of getting influenza vaccination (OR = 1.736; 95% CI = 1.151–2.618; *p* = 0.008). Women who had their pregnancy onset in autumn showed halved odds of getting influenza vaccination compared to women whose pregnancy onset was in spring (OR = 0.487; 95% CI = 0.287–0.828; *p* = 0.008). The chance of influenza vaccination uptake during pregnancy was around 38-fold increased when women had received a vaccination recommendation by HCW (OR = 37.767; 95% CI = 25.515–55.903; *p* < =0.001).

**Table 4:** Multivariable analysis of factors associated with receipt of provider recommendation for influenza vaccination and uptake of influenza vaccination

	Factors associated with receipt of provider recommendation for influenza vaccination	Factors associated with uptake of influenza vaccination
	OR; 95% CI; p	OR; 95% CI; p
Country of birth other than Germany	0.618; 0.408–0.937; 0.023	0.746; 0.380–1.463; 0.394
Marital status		
Divorced, widowed, living alone		0.744; 0.179–3.092; 0.684
Unmarried and living together		0.715; 0.428–1.195; 0.201
Married and living together		Ref.
Highest education^a^		
Low		0.806; 0.409–1.590; 0.534
Middle		0.699; 0.465–1.051; 0.085
High		Ref.
BMI (before pregnancy)		
Underweight		1.719; 0.535–5.528; 0.363
Normal weight		Ref.
Overweight		1.391; 0.950–2.038; 0.090
Health literacy		1.736; 1.151–2.618; 0.008
Receipt of provider recommendation		37.767; 25.515–55.903; <=0.001
Claiming of additional prenatal diagnostics		1.258; 0.787–2.012; 0.337
High-risk pregnancy	1.275; 1.005–1.618; 0.045	1.280; 0.886–1.847; 0.188
Season of pregnancy onset		
Spring (April, May, June)	Ref.	Ref.
Summer (July, August, September)	0.704; 0.515–0.962; 0.027	0.921; 0.570–1.489; 0.738
Autumn (October. November, December)	0.350; 0.250–0.490; <=0.001	0.487; 0.287–0.828; 0.008
Winter (January, February, March)	0.460; 0.330–0.642; <=0.001	0.925; 0.562–1.523; 0.759

^a^According to CASMIN-classification

### Trends

Comparing the frequency of vaccination recommendations by HCW during the three influenza seasons investigated in course of this study (namely season 2015/2016, season 2016/2017 and season 2017/2018) it could be shown that the frequency was continuously increasing. While in the season 2015/2016 16.6% of the women received a vaccination recommendation by their HCW, in the season 2016/2017 22.0% stated that their gynaecologist/obstetrician recommended them to get a flu shot and in the season 2017/18 the percentage continued to raise to 26.7%.

A similar trend was observed regarding influenza vaccination uptake. In season 2015/2016 11.0% of participating women had received influenza vaccination, in the season 2016/2017 13.5% and in the season 2017/2018 18.6%.

## Discussion

### Prevalence of receipt of recommendation and uptake of influenza vaccination

This study showed a low (13%) overall uptake rate of influenza vaccination by pregnant women over the study period 2015–2018 and also a low proportion of pregnant women who reported to have received a recommendation for the vaccination (20%). Considering the efficacy and safety of the vaccine, as well as the possible reduction of societal cost by preventing influenza infection in pregnant women [38, 39], an increase of this uptake rate should be urgently sought.

### Factors associated with receipt of provider recommendation

Being born in a country other than Germany was associated with a decreased chance of getting a vaccination recommendation by HCW. Providers might anticipate vaccination hesitancy in this group of women. The association between receiving a vaccination recommendation and pregnancy onset in spring might be explained by the fact these women’s second or third trimester of pregnancy (i.e. when influenza vaccination is recommended for every pregnant woman) is likely between November and February - months with increased risk of influenza infection and resulting greater provider awareness for vaccinations. At the same time providers treating women with high-risk pregnancy might be more aware of possible hazards during pregnancy and therefore more willing to recommend influenza vaccination.

In contrast to our findings a study which was conducted in the context of the national influenza immunization campaign in Germany in 2014 [40] found that 93% of gynaecologists/obstetricians advised their patients to get vaccinated against influenza during pregnancy. This discrepancy could result from an overrepresentation of HCWs with a positive attitude toward influenza vaccination in that study. At the same time women may be equally likely to underreport vaccination if the provider did not make a strong recommendation, if the woman was not receptive to the recommendation, or simply due to recall problems. Discrepancies between provider’s and patients’ reports regarding influenza vaccination recommendation and uptake during pregnancy were also found in a study from the U.S. [41]

Since influenza vaccination for pregnant women is officially recommended by the „German Standing Committee on Vaccination” (STIKO) [15, 16] in Germany, all gynaecologists/obstetricians are in charge to educate their pregnant patients about the possibility and need of being vaccinated against influenza. Given that there is no evidence that influenza vaccination is teratogenic [42], a change to the recommendations to vaccinate during the second and third trimester to make them consistent with the any-trimester recommendation for women at increased risk could result in better maternal vaccination rates in German women.

Reasons why providers currently do not adhere to the STIKO recommendation were investigated by Bödeker et al. The most frequently mentioned reasons reported by gynaecologists/obstetricians were safety concerns about the unborn child, high time consumption in informing patients about the recommendation, safety concerns about the pregnant women, and doubts about the efficiency of the vaccination [40]. Another recent study in Germany listed high requirement of time and effort to inform pregnant patients about vaccination as a barrier to recommending seasonal influenza vaccination [43]. A further study pointed out that physicians do not want to decide for pregnant women [44], possibly because they do not want to be liable for any adverse events.

### Factors associated with uptake of influenza vaccination

We found that increasing health literacy goes along with a higher chance of getting influenza vaccination and women whose pregnancy onset was in autumn showed lower odds of receiving influenza vaccination compared to women with pregnancy onset in spring. However, with an OR of 38 the strongest association with receipt of influenza vaccination was seen for having received vaccination recommendation by HCW. The presence of recommendations by HCW was found to be a strong influencing factor on influenza vaccination uptake during pregnancy also in studies from Australia and the United States [30, 45, 46].

### Comparison of associated factors based on Andersen’s model

Our study was based on the theoretical framework of Andersen’s behavioural model of health services. While the receipt of vaccination recommendation was mainly influenced by ‘predisposing characteristics’, with the variable country of birth and ‘need’ with the variables high-risk pregnancy and season of pregnancy onset, the uptake of vaccination was affected by the domains ‘need’ with the season of pregnancy onset and ‘enabling resources’ with the variables health literacy and receipt of vaccination recommendation.

### Strengths and limitations of the study

A strength of the present study is its large sample size leading to a higher statistical power. Another strength of this study is the consideration of many different variables and thereby the availability of a comprehensive data record, out of which many potential influencing factors could be analysed.

Our study has some limitations that need to be addressed. Out of the total study enrolment with a number of 2620 participants, only 1886 women could be included in final statistical analysis. This might weaken the representativeness of the study population. Furthermore, women with high school degree are overrepresented. This phenomenon is known from other surveys, where women with higher education level or socioeconomic standing are more likely willing to participate in studies like that [47]. Moreover, the exclusion of women with insufficient German language skills might reduce its external validity. However, the study team tried to include all women whose German language skills were sufficient to participate. Another limitation is the fact that we had to rely on self-reported data, both for predictor and outcome variables. We were not able to verify them with medical records or similar objective data collection with a thus arising potential of recall bias. Possibly, recommendation was only reminded by women, who got vaccinated against influenza subsequently and socially desirable responses are given preferentially. However, previous studies suggest that self-reported information about having received or not received influenza vaccination can be considered reliable [48, 49]. As a further limitation we need to address that we might have missed women who were vaccinated prior to pregnancy onset, but within the same season. This might have led to an underestimation of number of women who got vaccinated, as these women were not eligible for vaccination during pregnancy. Since there is no recommendation for influenza vaccination for young healthy women in Germany, unless they are working in the medical field, we assume that the vast majority of women did not receive influenza vaccination before pregnancy.

## Conclusions

This study showed that influenza vaccination coverage for pregnant women is low. Lack of vaccination recommendations by HCW can be considered the main reason, as only 20,3% of women received the recommendation while it was simultaneously found to be the strongest influencing factor on vaccination uptake by far.

Efforts should be made to increase the number of HCWs who recommend the influenza vaccination to their pregnant clients. On the one hand, awareness among gynaecologists/obstetricians towards the importance of influenza vaccination during pregnancy must be raised. On the other hand, gynaecologists/obstetricians should also be enabled and supported to inform their patients properly about vaccination, e.g., in terms of providing them with information material or guidelines for communication with vaccination scepticism.

## Supplementary Information

**Additional file 1.** Supplementary Information.

## Data Availability

The datasets used and analysed during the current study are available from the corresponding author on reasonable request.

## Abbreviation

HCW: Health care worker
