# Supplementary material for: Prevalence of and factors associated with receipt of provider recommendation for influenza vaccination and uptake of influenza vaccination during pregnancy: cross-sectional study
Source: BMC Pregnancy Childbirth. 2021 Oct 27;21:723. doi: 10.1186/s12884-021-04182-w (PMC8549148; doi:10.1186/s12884-021-04182-w)
Supplement: Supplementary file 1 — Additional file 1. Supplementary Information. [file 12884_2021_4182_MOESM1_ESM.docx]

**Supplementary Information**

*KUNO-Kids Study group:*

Andreas Ambrosch ^6^, Petra Arndt^7^, Andrea Baessler^8^, Mark Berneburg^9^, Stephan Böse-O’Reilly^10^, Romuald Brunner^11^, Wolfgang Buchalla^12^, Sara Fill Malfertheiner^3^, André
Franke^13^, Sebastian Häusler^3^, Iris Heid^14^, Caroline Herr^2^, Wolfgang Högler^15^, Sebastian Kerzel^1^, Michael Koller^16^, Michael Leitzmann^17^, David
Rothfuß^18^, Wolfgang Rösch^19^, Bianca Schaub^20^, Bernhard H.F. Weber^21^, Stephan Weidinger^22^, Sven Wellmann^1^

*6) Institute of Laboratory Medicine, Microbiology and Hygiene, Barmherzige Brüder Hospital, Regensburg, Germany*

*7) ZNL Transfercenter of Neuroscience and Learning, University of Ulm, Ulm, Germany*

*8) Department of Internal Medicine II, Regensburg University Medical Center, Regensburg, Germany*

*9) Department of Dermatology, University Medical Centre Regensburg, Regensburg, Germany*

*10) Institute and Clinic for Occupational, Social and Environmental Medicine, University Hospital, LMU Munich, Munich, Germany*

*11) Clinic of Child and Adolescent Psychiatry, Psychosomatics and Psychotherapy, Bezirksklinikum Regensburg (medbo), Regensburg, Germany*

*12) Department of Conservative Dentistry and Periodontology, University Hospital Regensburg, University of Regensburg, Regensburg, Germany*

*13) Institute of Clinical Molecular Biology, Christian-Albrechts-University of Kiel, Kiel, Germany*

*14) Department of Genetic Epidemiology, University of Regensburg, Regensburg,*

*Germany*

*15) Department of Pediatrics and Adolescent Medicine, Johannes Kepler University Linz, Linz, Austria*

*16) Center for Clinical Studies, University Hospital Regensburg, Regensburg, Germany*

*17) Department of Epidemiology and Preventive Medicine, University of Regensburg, Regensburg, Germany*

*18) City of Regensburg, Coordinating Center for Early Interventions, Regensburg, Germany*

*19) Department of Pediatric Urology, University Medical Center, Regensburg, Germany*

*20) Pediatric Allergology, Department of Pediatrics, Dr. von Hauner Children's Hospital, University Hospital, LMU Munich, Munich, Germany*

*21) Institute of Human Genetics, University of Regensburg, Regensburg, Germany*

*22) Department of Dermatology, Venereology and Allergy, University Hospital Schleswig-Holstein, Campus Kiel, Kiel, Germany*
